# Electrical Stimulation of Acute Fractures: A Narrative Review of Stimulation Protocols and Device Specifications

**DOI:** 10.3389/fbioe.2022.879187

**Published:** 2022-06-02

**Authors:** Peter J. Nicksic, D’Andrea T. Donnelly, Nishant Verma, Allison J. Setiz, Andrew J. Shoffstall, Kip A. Ludwig, Aaron M. Dingle, Samuel O. Poore

**Affiliations:** ^1^ Division of Plastic Surgery, University of Wisconsin School of Medicine and Public Health, Madison, WI, United States; ^2^ Department of Biomedical Engineering, University of Wisconsin—Madison, Madison, WI, United States; ^3^ Wisconsin Institute for Translational Neuroengineering (WITNe), University of Wisconsin—Madison, Madison, WI, United States; ^4^ Department of Biomedical Engineering, Case Western Reserve University, Cleveland, OH, United States; ^5^ APT Center, Louis Stokes Cleveland VA Medical Center, Cleveland, OH, United States; ^6^ Department of Neurological Surgery, University of Wisconsin—Madison, Madison, WI, United States

**Keywords:** electrical stimulation, fracture, human, direct current, pulsed electromagnetic field, capacitive coupling

## Abstract

Orthopedic fractures have a significant impact on patients in the form of economic loss and functional impairment. Beyond the standard methods of reduction and fixation, one adjunct that has been explored since the late 1970s is electrical stimulation. Despite robust evidence for efficacy in the preclinical arena, human trials have mixed results, and this technology is not widely accepted. The purpose of this review is to examine the body of literature supporting electrical stimulation for the purpose of fracture healing in humans with an emphasis on device specifications and stimulation protocols and delineate a minimum reporting checklist for future studies of this type. We have isolated 12 studies that pertain to the administration of electrical stimulation for the purpose of augmenting fracture healing in humans. Of these, one was a direct current electrical stimulation study. Six studies utilized pulsed electromagnetic field therapy and five used capacitive coupling. When examining these studies, the device specifications were heterogenous and often incomplete in what they reported, which rendered studies unrepeatable. The stimulation protocols also varied greatly study to study. To demonstrate efficacy of electrical stimulation for fractures, the authors recommend isolating a fracture type that is prone to nonunion to maximize the electrical stimulation effect, a homogenous study population so as to not dilute the effect of electrical stimulation, and increasing scientific rigor in the form of pre-registration, blinding, and sham controls. Finally, we introduce the critical components of minimum device specification reporting for repeatability of studies of this type.

## 1 Introduction

Traumatic fractures are estimated to incur $265.4 billion dollars per year of economic loss due to cost of healthcare and time away from work in the United States alone (Yelin et al., 2016). Additionally, delays in fracture healing are associated with increased pain, functional limitations, and a decreased quality of life for patients (Cook et al., 2015). For these reasons, many efforts have been made to augment and expedite the process of fracture healing. These types of adjunct therapies include electrical stimulation (ES) and low-intensity pulsed ultrasound therapy (LIPUS). A recent meta-analysis aggregated 13 randomized controlled trials examining the effect of LIPUS (10) and pulsed electromagnetic field (PEMF) therapy (3) on acute fracture healing (Hannemann et al., 2014a). While there was no significant effect for time to radiographic union overall, subgroup analysis found that these treatments decreased time radiographic union for subgroups of upper extremity fractures (mean difference = −20.23 days, 95% CI −32.68– −7.77, *p* = 0.001, I^2^ = 67%) and non-operative fractures (mean difference = −26.65 days, 95% CI = −50.38– −2.91, *p* = 0.03, I^2^ = 98%) (Hannemann et al., 2014a). While LIPUS and other adjuncts may show promise in improving outcomes in specific subtypes of acute fractures, there is large body of literature in support of ES for the purpose of bone healing. Electrical stimulation and its relationship to bone formation was first described by Fukada and Yasuda (1957). In their seminal paper, they described the piezoelectric quality of bone—that is, that bone generates endogenous electrical fields when put under mechanical stress. Since then, researchers have attempted to harness exogenous ES to heal a variety of osseous insults in both animals and humans.

There are three modalities of ES currently in use: direct current electrical stimulation (DCES), capacitive coupling (CC), and inductive coupling. Direct current electrical stimulation (DCES) is an invasive method in which the cathode is placed percutaneously directly into the site of osseous injury and electron flow is unidirectional from the anode, which is placed in nearby subcutaneous tissue. Capacitive coupling is most commonly noninvasive and involves electrodes which are placed on the skin on either side of osseous injury. The alternating current generates an electrical field between the electrodes. Inductive coupling, most commonly in the form of PEMF, utilizes two solenoids oriented parallel to the skin surface on opposite sides of the osseous injury. Current is pulsed through the solenoids, and a magnetic field is generated, which induces a perpendicular electrical field.

There is a robust body of evidence in support of ES for fracture non-unions, spinal fusions, and acute fractures in small animal models (Guizzardi et al., 1994; France et al., 2001; Atalay et al., 2015; Liu et al., 2021; Nicksic et al., 2022). There has also been a resurgence in interest in the field of ES for the purpose of bone healing in *in vitro* studies (Li et al., 2019; Portan et al., 2019; Escobar et al., 2020; Stephan et al., 2020). However, when translating these stimulation protocols and device specifications to large animals and humans, there has been varying levels of success (Paterson et al., 1977a; Paterson et al., 1977b; Miller et al., 1984; Law et al., 1985; Muttini et al., 2014).

The reason there has been mixed success in the translation of ES for bone healing from *in vitro* and small animal studies to large animal studies and clinical trials is likely multifactorial. One issue likely lies in getting stimulation to the fracture site in larger limbs. For the more popular noninvasive stimulation modalities, computer modeling data demonstrates that changing the thickness of skin, subcutaneous fat, muscle, and cortical bone all have significant effects on the energy transmitted to the fracture site (Lunt, 1985; Plonsey and Barr, 1995). This is supported by the fact that large animal studies using DCES—where stimulation can be administered reliably regardless of scale—are largely more successful in inducing osteogenesis than noninvasive treatments (Nicksic et al., 2022).

A recent meta-analysis of randomized, sham-controlled clinical trials demonstrated that electrical bone growth stimulators (EGBS) are only effective at reducing the 12 months non-union rate in the spinal fusion subgroup (*p* = 0.002, 95% CI = 0.45–0.84), and the effect for acute fractures, non-union, delayed union, and osteotomy subgroups was not significant (Aleem et al., 2016). One indication for the use of ES is the treatment of acute (fresh) fractures—fractures expected to heal within the normal time course for the injury once promptly and adequately reduced and fixated—which are the most common osseous injury (Yelin et al., 2016). Returning to the literature to examine the methodologies of human studies for acute fractures may shed light on why ES is not a widely accepted therapy.

Given the abundance of evidence in preclinical studies and mixed results from clinical trials, this review aims to 1) summarize the data of key studies for the treatment of acute fractures with ES stratified by ES modality, 2) examine the methodologies of these studies with attention to ES device specifications and stimulation protocols, and 3) draw conclusions about what is necessary to conduct a future study of this type so that the methodology may be applied to other fracture types. To do this, we queried the MEDLINE database for relevant search terms including combinations of “electrical stimulation,” “bone healing,” “fracture,” “direct current,” “PEMF,” and “capacitive coupling,” searching for human studies pertaining to the treatment of acute fractures with electrical stimulation. *In vitro* and animal studies, case reports, and studies not available in the English language were excluded. Our query yielded one DCES study, six PEMF studies, and five CC studies (Table 1).

**TABLE 1:** This table presents the pre-registration, randomization, sham-control, and blinding status of the studies included in our review, organized in chronological order. This is followed by number of participants, details of the study population, type of fracture, stimulation protocol, device specifications, and outcomes of the studies. Green fill denotes significant results. Red fill denotes nonsignificant results. Gray fill denotes that the study results are not applicable.

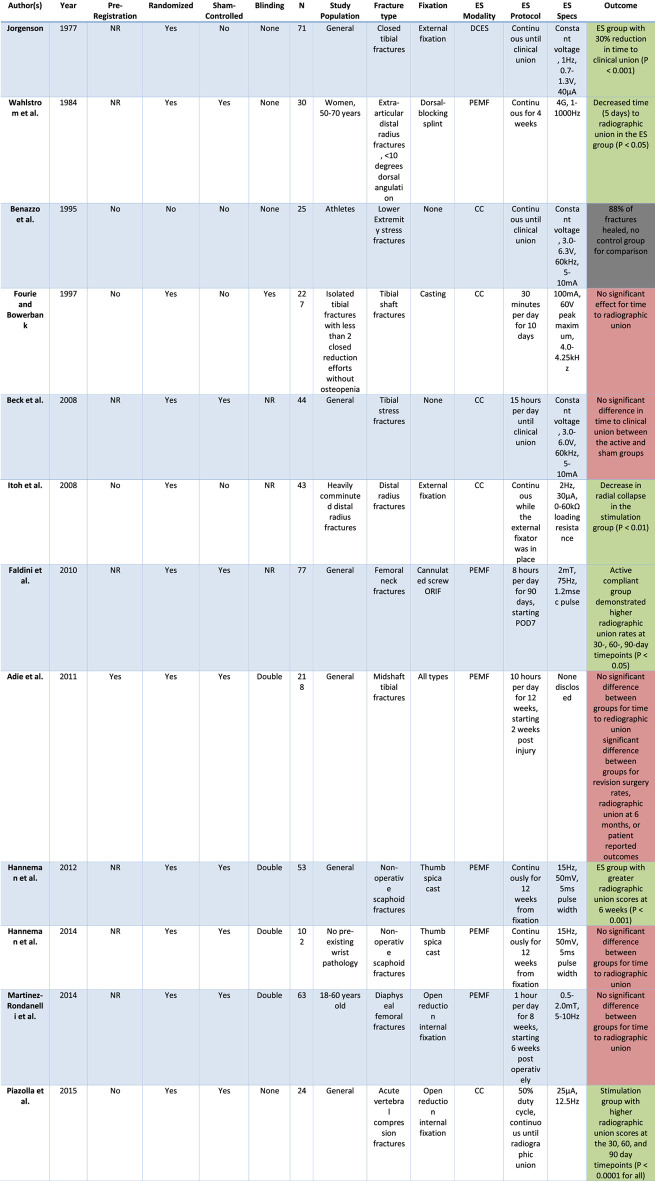

N, number of participants; ES Specs, electrical stimulation specifications.

## 2 Factors Affecting Fracture Healing

When evaluating orthopedic fractures, there are many factors intrinsic to the injury pattern that can give the clinician an estimate of the healing potential and level of intervention required. One important factor affecting fracture healing is fracture location. Generally, time to radiographic union (when the bone is considered healed on radiographs or computed tomography) and/or clinical union (when the patient can resume normal function without pain or limitation) is inversely proportional to the vascular supply of the bone. For example, the mid-diaphysis of the femur has a robust vascular supply from the surrounding muscle, and typically heals relatively quickly and reliably. By contrast, the femoral neck and mid-diaphyseal tibia have minimal surrounding vascularized tissue, and fractures of these locations are often treated more aggressively as the healing potential is lower (Zura et al., 2016b). A similar concept holds true for “short bones”—the carpal and tarsal bones. These bones have a tenuous vascular supply and are prone to nonunion (Zura et al., 2016b). One other important intrinsic factor that affects fracture healing capacity is the level of comminution. Highly comminuted fractures—or fractures with multiple small fragments—typically are associated with higher energy injuries or lower bone quality. Speaking broadly, a fracture with a higher level of comminution is more likely to proceed to malunion (a fracture that heals in an extra-anatomic position) or nonunion. For example, a minimally comminuted distal radius fracture may be treated with a thumb spica cast, whereas a more comminuted distal radius fracture might require open reduction, possibly bone grafting, and plating to heal (Ring et al., 2005).

In addition to factors intrinsic to the injury itself, there are patient-related factors that can greatly affect fracture healing. A recent meta-analysis aggregated all patient factors associated with nonunion (Zura et al., 2016a). In this study, they demonstrated that advanced patient age, smoking history, alcoholism, osteoporosis, obesity, and diabetes were all associated with increased rates of nonunion for various fracture locations (Zura et al., 2016a). Between male and female sex, the nonunion rate has been shown to be higher in women after the age of 45–54 years. However, younger women have a lower rate of operative nonunions than men (Mills et al., 2017). All of these factors are important to consider when analyzing results from studies utilizing ES to augment fracture healing.

## 3 Direct Current Electrical Stimulation

**FIGURE 1:**
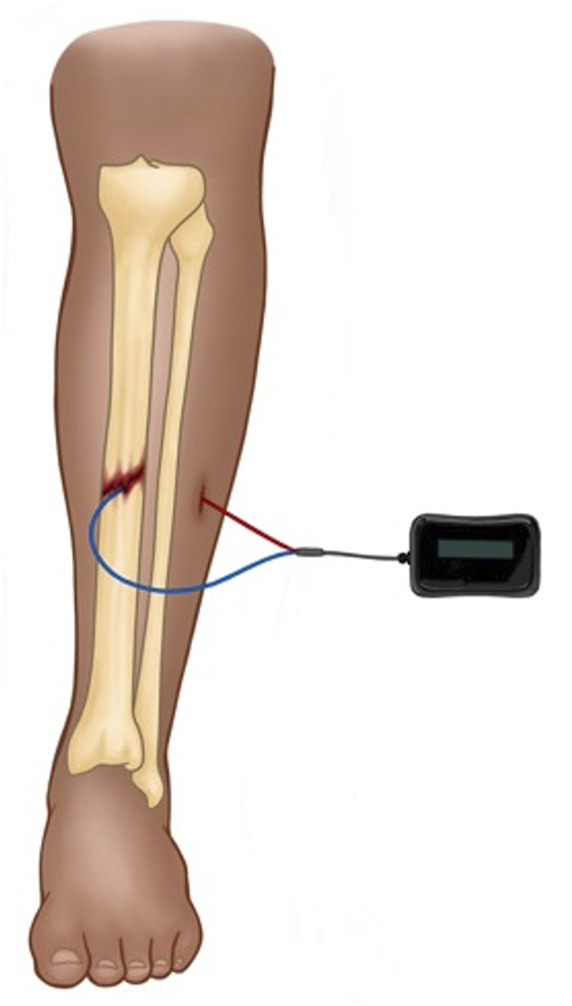
Depicted is a schematic for DCES in the treatment of a midshaft tibial fracture. The device is comprised of a power source (which is typically placed subcutaneously) and the cathode (blue lead) and anode (red lead). The cathode is placed at the fracture site, and the anode is placed in nearby soft tissue.

Direct current electrical stimulation involves percutaneous wire leads placed into the area of osseous injury (Figure 1). The cathode placed at the site of osseous injury results in electrochemical reduction of molecular oxygen, which is believed to create an alkaline, low-oxygen microenvironment (Brighton and Friedenberg, 1974; Haddad et al., 2007). This state favors osteoblast differentiation and stimulates osteoclasts to produce vascular endothelial growth factor, which induces vasculogensis (Bushinsky, 1996; Kaysinger and Ramp, 1998). Because lead placement requires an operation and carries risk of infection or device malfunction, DCES is not an optimal modality for treatment of acute fractures. As such, DCES is typically reserved for occasional use in operative, acute fractures with high-risk of delayed union or non-union, as many surgeons would prefer the standard methods of reduction and fixation first before accepting the infection risk associated with lead placement and removal. Another shortcoming of DCES is that, because the flow electrons is unidirectional, the cathode accumulates a negative charge at higher currents, leading to the deleterious concentrations of faradic salts (Zengo et al., 1976). Therefore, there is a limit to the current density and charge that can applied safely (Brighton et al., 1981).

### 3.1 DCES May Be Effective for Fracture Healing but With a Significant Infection Risk

However, one randomized study was performed that examined DCES treatment in 71 patients with closed, acute tibial fractures treated with an external fixator (Jorgensen, 1977). There was no mention of pre-registration in the manuscript. Of these, 12 were excluded for lead-site infections. There were no other inclusion or exclusion criteria for fracture morphology or injury type defined. Insulated screws proximal and distal to the fracture acted as the cathode, but the screw material was not stated. Additionally, the screws were not insulated from the skin or muscle, and no current measurement at the level of the bone was performed. The experimental group was treated with a voltage-controlled constant stimulation of 1 Hz, and 0.7–1.3 V with a measured current of 40 μA until the fractures met criteria for clinical union, as defined by lack of fracture mobility on clinical exam and pain-free ambulation. Participants underwent monthly radiographs and mechanical testing until the fracture attained a set degree of stiffness. The experimental group showed an accelerated time to clinical union by 30% (2.4 months) when compared to controls (3.6 months) (*p* < 0.001).

## 4 Pulsed Electromagnetic Field

**FIGURE 2:**
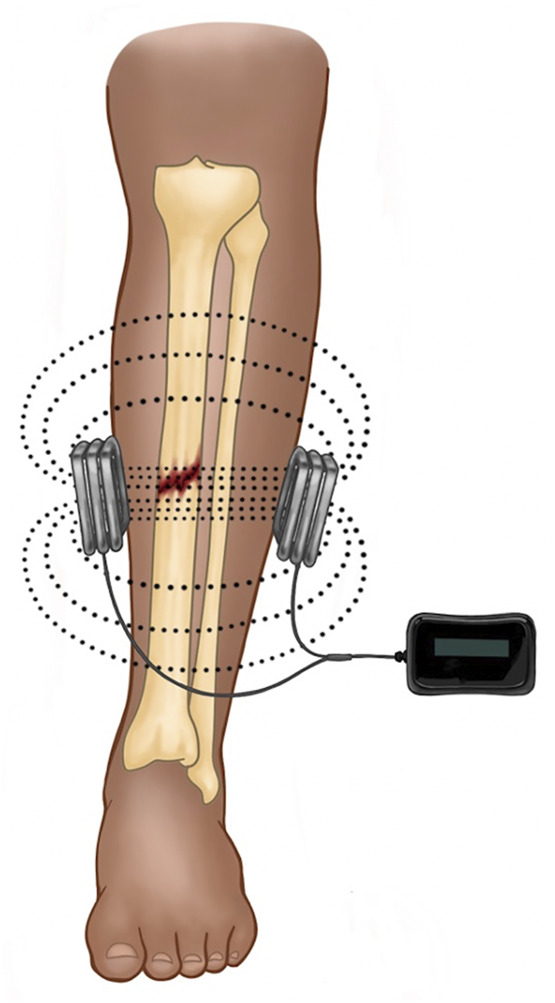
Depicted is a schematic for PEMF in the treatment of a midshaft tibial fracture. The device is completely external and comprised of a power source and two solenoids oriented parallel to the skin surface. Current is pulsed through the solenoids and a magnetic field is produced (dotted lines), which induces a perpendicular electrical field (not depicted).

Pulsed electromagnetic field therapy is a more attractive modality to augment acute fracture healing, because it can be administered non-invasively and, therefore, does not carry risks of infection or additional operations (Figure 2). Pulsed electromagnetic field therapy is believed to increase cytoplasmic calcium by activating voltage-gated calcium channels intracellularly and is associated with upregulation of multiple key growth factors (insulin-like growth factor 2, bone morphogenic proteins [BMPs] 2 and 4, and transforming growth factor -beta {TGF-β}) inducing osteoblast differentiation, proliferation, and extracellular matrix deposition (Haddad et al., 2007; Khalifeh et al., 2018).

### 4.1 PEMF Demonstrates Mixed Results for Long Bone Fracture Healing With Compliance Issues

Studies have examined the effect of PEMF therapy on healing of long bone fractures prone to delayed or nonunion. Wahlström (1984) published an unblinded, randomized, sham-controlled study evaluating the effect of PEMF on 30 women between the ages of 50–70 years with acute, non-operative, extra-articular distal radius fractures with apex dorsal angulation less than 10 degrees. No mention of pre-registration was made. Post-injury day one, participants were fixed with a dorsal-blocking plaster cast with an imbedded copper solenoid and current generator, producing a magnetic field of 4 Gauss (G) within the range of 1–1000 Hz for 4 weeks. No solenoid geometry was reported. The apparatus was checked weekly with a gaussmeter to ensure continued functioning, but no calculations or measurements were offered to estimate the magnetic field at the level of the fracture. Bone scintigraphy and radiographs were completed at 1, 2, 4 and 8 weeks to evaluate for fracture displacement and radiographic union. Radionucleotide uptake ratio—the intensity of the callus on bone scintigraphy referenced to a non-healing control bone—was significantly higher in the stimulation group at week 1 and 2 (*p* < 0.05, *p* < 0.01 respectively). There was no significant difference at 4 and 8 weeks. Time to radiographic union, defined as when the intensity of radionucleotide uptake started to decrease, was significantly reduced in the stimulation group at 18 days versus 23 days in the control group (*p* < 0.05). Of note, the rate of re-displacement of fractures was nonsignificant between the treatment and control groups and was instead correlated to original displacement and stability of reduction.

Faldini et al. (2010) investigated the use of PEMF on operative fractures of the femoral neck in 77 patients, randomized to either sham or active stimulation. No mention of pre-registration was made. The fractures were fixed percutaneously with cannulated screws within 3 days post-injury, and stimulation with sham versus active PEMF devices began on post-operative day 7. The PEMF device used was the Biostim (Igea, Carpi, Italy), and the stimulation protocol consisted of 2 mT peak magnetic field, 75 Hz, and 1.2 msec pulse duration for 8 h per day for 90 days. Specific coil dimensions were not reported. Compliance was monitored with a timer inside the device that recorded device activity. Radiographs were completed at regular intervals to 2 years following surgery. Radiographic union was defined as a minimum of 70% of the fracture being bridged with trabeculae. The level of compliance was heterogenous in both the active and control groups, so data analysis was performed in subgroups of compliant (greater than 6 h per day of device use) and noncompliant (less than 6 h per day of device use). Patients in the active compliant group demonstrated a higher rate of radiographic union at the 30-, 60-, and 90-days timepoints when compared to the active noncompliant and placebo groups (*p* < 0.05). Of note, the active compliant group was significantly younger than the active noncompliant group (67.1 ± 6.0 years, 71.6 ± 3.1 years, respectively, *p* < 0.05).

Martinez-Rondanelli et al. (2014) examined the effect of PEMF on closed, diaphyseal femoral fractures in a double-blind, randomized, sham-controlled study of 63 patients, 18–60 years of age. No mention of pre-registration was made. Custom stimulation devices delivering 5–10 Hz and 0.5–2.0 mT were applied 6 weeks after operative fixation of the fractures, 1 hour per day for 8 weeks. Depending upon the patients’ thigh diameter, the coil diameters could be adjusted, but no calculations were offered as to how this was performed, and no coil geometry was described. Radiographs were performed at 12, 18, and 24 weeks post-operatively and were classified by a radiologist as non-union, partial union, or complete union. There was no significant difference in rate of radiographic union between groups.

Adie et al. (2011) performed a pre-registered, randomized, double-blind, sham-controlled trial for PEMF on 218 tibial diaphyseal fractures. All midshaft tibial fractures were included, regardless of means of fixation. This study used the commercially available Biomet^®^ Bone Healing System (Zimmer Biomet, Indiana, IN, United States) PEMF device, and the device specifications were not disclosed. The device was applied over the plaster or fiberglass cast for 10 h per day for 12 weeks, 2 weeks following injury and initial fixation. Compliance was measured by the device’s internal timer, which clocked hours of use. Outcomes measures included rate of secondary surgery for nonunion at 1-year post-injury, radiographic union at 6 months, and patient reported outcomes at 1 year. In this study, there were no significant effects of PEMF for revision surgery rates, radiographic union at 6 months, or patient reported functional outcomes.

### 4.2 Little Efficacy Demonstrated With PEMF for Healing Short Bone Fractures

In addition to long bone fractures, there have been PEMF studies on fractures of short bones prone to nonunion and delayed union. Hannemann et al. (2012) conducted a double-blind, sham-controlled study in which the effect of PEMF was examined in 53 nonoperative scaphoid fractures. No mention of pre-registration was made. All patients had thumb spica cast-implanted PEMF stimulators (Ossatec, Uden, Netherlands) with only the treatment group being active. The device specifications were 15 Hz, 50 mV, and a pulse width of 5 μs and burst width of 5 ms. No coil geometry or magnetic field strength was reported. Stimulation was applied continuously until complete union or 12 weeks post-injury. Radiographic union was blindly assessed by two surgeons and a radiologist and categorized into three groups: nonunion, possible union, and complete union. Tenderness at the anatomic snuffbox was assessed with longitudinal compression of the scaphoid. Clinical exams and radiographs were obtained at regular intervals to 1-year post-injury. The treatment group demonstrated a reduction in snuffbox tenderness (*p* = 0.003) and increased radiographic union scores (*p* < 0.001) at the 6-week timepoint only.

Hannemann et al. (2014b) performed a similar study evaluating PEMF for nonoperative scaphoid fractures in 102 patients using computed tomography (CT) imaging instead of plain radiographs. There was no mention of pre-registration. Patients were excluded if their fractures were displaced, if they had proximal pole scaphoid fractures, fracture dislocations, additional fractures, or pre-existing wrist impairments. Patients received continuous stimulation with the same device (Ossatec, Uden, Netherlands) incorporated into a plaster cast placed above the fracture site with the same specifications as the previous study (Hannemann et al., 2012). Clinical and CT examinations were performed at regular intervals up to 1 year. There was no significant difference in time to radiographic union between groups.

## 5 Capacitive Coupling

**FIGURE 3:**
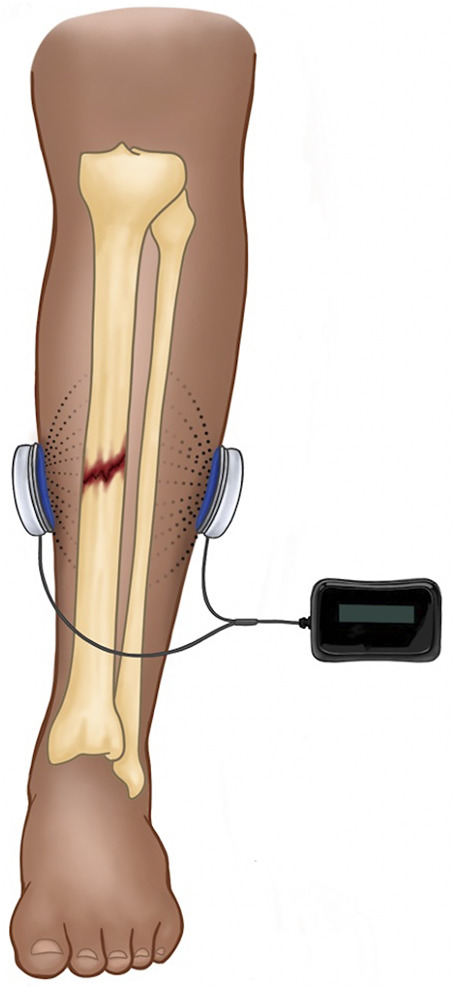
Depicted is a schematic for CC in the treatment of a midshaft tibial fracture. The device is completely external and comprised of a power source and two skin surface electrodes. The alternating current generates an electrical field between the electrodes (dotted lines) with significant falloff into tissue (dissipation of dotted lines).

Capacitive coupling is typically a non-invasive method of ES in which electrodes are placed on the skin, and an alternating current generates an electrical field between the electrodes (Figure 3). Similar to PEMF, CC induces an increase in cytoplasmic calcium by activating voltage-gated calcium channels, which initiates the calmodulin-mediated pathway of osteogenesis (Crocker et al., 1988; Brighton et al., 2001). Capacitive coupling therapy is also associated with upregulation of BMP 2 and 4 and TGF-β1 in osteoblasts (Zhuang et al., 1997; Wang et al., 2006). With alternating current, there is no risk of accumulation of faradic salts at the electrode/electrolyte interface, because the electrochemical reactions are persistently being reversed. This allows for much higher charge densities than would be possible with direct current. Due to poor penetrance of the electrical field into soft tissue (Plonsey and Barr, 1995), CC is only practical for superficial bones (e.g., distal radius or tibia).

### 5.1 CC may be Useful in the Treatment of Lower Extremity Stress Fractures

Benazzo et al.(1995) performed an open case-series (therefore, no control group) of tarsal, metatarsal, fibular and tibial stress fractures of 25 athletes treated with CC. The device provided a voltage-controlled sinusoidal waveform from 3.0 to 6.3 V, and 60 kHz. The current was estimated to be between 5 and 10 mA. No electrode geometry or spacing was provided to calculate charge density. Stimulation was continuous until the fracture demonstrated radiographic evidence of healing or until 3 weeks passed with no reported change. Participants underwent bi-weekly radiographic evaluation. Fractures were determined to be healed if the radiographs showed incomplete healing with a marked decrease of pain. Final radiological assessment found that 88% of fractures healed, 8% improved and 4% did not heal. Beck et al. (2008) also examined the effect of CC on 44 patients with tibial stress fractures in a randomized, sham-controlled study. No mention of pre-registration was made. Constant voltage stimulation of 3.0–6.0 V, 60 kHz with a measured 5–10 mA was applied for 15 h a day until the fractures reached clinical union. No electrode geometry or spacing was provided to calculate charge density. Clinical union was determined by the absence of reported pain after hopping 10 cm for 30 s. There was no significant difference in time to clinical union between the active and sham groups, however superior treatment compliance was associated with reduced healing time in the experimental group (*p* = 0.003).

### 5.2 CC With Mixed Results for Long and Short Bone Fracture Healing at a Wide Range of Stimulation Parameters

Fourie and Bowerbank applied CC to acute tibial fractures of 227 patients in an unregistered, blinded, randomized study without sham controls (Fourie and Bowerbank, 1997). Patients were excluded if they had additional fractures, associated medical comorbidities, or if they received two or more closed reduction efforts. All mechanisms of injury, fracture morphologies, and fracture locations within the tibia were included. Electrodes were placed in windows of a plaster cast with the guidance of radiographs, delivering constant voltage stimulation with an estimated 100 mA and a 60 V peak maximum. One subgroup received 4.0 kHz, while another subgroup received 4.10–4.25 kHz. No electrode geometry or spacing was provided to calculate charge density, and no measurements of electric field were offered. The fractures were stimulated 30 min per day for 10 days. Radiographic union was assessed by a single-blinded radiologist, and the fractures were categorized into non-union, partial union, and nonunion. There were no significant differences in time to radiographic union between groups. However, there was a significant effect of time to radiographic union when fractures from both stimulation and control groups were analyzed by mechanism of injury (*p* = 0.0005), type of fracture (*p* = 0.015), the presence of a fractured fibula (*p* = 0.026), and degree of displacement (*p* = 0.0005) for time to radiographic union.

Itoh et al. (2008) investigated CC on heavily comminuted, intra-articular fractures of the distal radius in 43 patients in a randomized study without sham controls. No mention of blinding was made. The pins of an external fixator acted as the electrodes and provided a 2 Hz sinusoidal wave of constant current stimulation at 30 μA with 0–60 kΩ of loading resistance. No electrode geometry or spacing was provided to calculate charge density, and no measurements of electric field were offered. A control group received an external fixator with no electrical stimulation. The fractures were considered healed when radiographs showed the presence of bridging trabeculae, and no remaining radiolucent lines between fracture fragments. Radiographs completed weekly demonstrated a significant decrease in radial collapse in the stimulation group (*p* < 0.01). Results of this study must be guarded, however, because the external fixators were left in place for an average of 39.0 ± 4.2 and 48.9 ± 7.2 weeks for the control and stimulation groups, respectively, which is significantly longer than what is considered standard of care for duration of external fixator placement.

In 2015, Piazzolla et al. (2015) evaluated the efficacy of CC on 24 acute vertebral compression fractures in an unregistered, randomized, sham-controlled study. The electrodes placed on the skin delivered constant current stimulation at 25 μA at 12.5 Hz with a duty cycle of 50%. No electric field measurements or electrode geometry or spacing were reported. Magnetic resonance imaging (MRI) was completed at 0, 30, 60 and 90 days. An unblinded neuroradiologist and two orthopedists analyzed the MRIs to determine healing scores. The stimulation group demonstrated higher radiographic healing scores at the 30-, 60- and 90-day timepoints (*p* < 0.0002, *p* < 0.0001, and *p* < 0.0001, respectively). There was an improvement in pain scores and patient reported outcomes as determined by the Visual Analog Scale, and Oswestry Low Back Disability Index (*p* = 0.007, *p* = 0.002, respectively). Complete healing measured by MRI was found only at the 90-day timepoint in the stimulation group (*p* = 0.0001).

## 6 Discussion

### 6.1 Optimizing Outcomes in EGBS Studies for Acute Fractures

This extensive examination of the body of literature of ES on osseous healing has revealed several important trends. The first issue presents itself in selecting the study population. Ideally, the study population should be a group of patients who would benefit most from EGBS therapy. The fracture location should be an area with a high rate of nonunion (e.g., middle tibia or scaphoid) (Zura et al., 2016b) and strict inclusion and exclusion criteria should be applied so that the effect of ES is not diluted. This includes controlling for multiple variables like fracture morphology, mechanisms of injury, patient comorbidities, smoking status, sexes, and ages for a given fracture location. For example, Fourie and Bowerbank (1997) examined the effect of CC on tibial fractures and found no effect based on ES but did find significant correlations for mechanism of injury, fracture morphology, degree of displacement, and presence of fibular fractures. If the study population, for example, consisted of osteopenic females aged 54–70 years with minimally displaced distal radius fractures from a fall from standing, the chances of a significant effect for ES may be larger.

Another common issue is the lack of consistency in reporting of device specifications. Many studies devise custom stimulators and do not offer any calculation or measurement of the strength of stimulation. As such, the study is not replicable, and any results of the study cannot be applied to other fracture locations or patient populations. For DCES studies, we would recommend reporting the current density (measured or calculated), frequency, and cathode material, as these variables have all been shown to affect osteogenesis in animal studies (Spadaro and Becker, 1979; Spadaro, 1982). For CC studies, we would recommend reporting electrical field strength (measured) or enough information (electrode geometry and distance between them) to calculate charge density, frequency, and distance between electrodes. For PEMF studies, the magnetic field strength (measured or calculated), frequency, and coil geometry (number of turns and size and shape of the solenoid) are necessary to replicate results.

Finally, we recommend National Institute of Health pre-registration of future EGBS studies with an overall increase in methodological rigor. Ideally, studies would be double-blinded and sham-controlled with statistical power analysis performed based on anticipated effect size. The stimulation protocol – hours of stimulation per day and weeks of therapy duration – would be derived from past studies and be justified with data. Currently, stimulation protocols are heterogenous for any given modality. For example, PEMF studies range from 1 hour per day for 8 weeks to constantly for 12 weeks (Hannemann et al., 2012; Hannemann et al., 2014b; Martinez-Rondanelli et al., 2014). These studies should also define strict criteria or a quantifiable metric for endpoints like radiographic or clinical union. Standardizing these variables and outcome measures would provide meaningful and replicative data that would potentially move ES into the clinical arena should positive results be found.

### 6.2 The Optimal ES Modality for Treatment of Acute Fractures

When determining what the most promising ES modality is for the treatment of acute fractures, each of the available modalities – DCES, CC, and PEMF – have significant advantages and weaknesses. The ideal EGBS would possess qualities of each of these modalities. Direct current electrical stimulation can be delivered directly to the fracture site, bypassing intervening soft tissue and offering a predictable amount of current to treat the fracture (Figure 1). When examining the large animal literature, DCES studies are typical more successful in inducing osteogenesis than the noninvasive modalities (Nerubay et al., 1986; Chakkalakal et al., 1990; Cook et al., 2004). These studies applied a wide range of stimulation currents and were successful in inducing osteogenesis within a range of 20–100 μA, and lower stimulation currents (13 μA) were not successful (Nerubay et al., 1986; Chakkalakal et al., 1990; Toth et al., 2000). These results are problematic for translation into humans, however, because these studies used custom-built stimulators and did not report cathode material, lead geometry, or measures of current density in all cases (Nicksic et al., 2022). Direct current electrical stimulation remains largely unstudied in humans as it is impractical, requiring operations for lead placement and removal and presenting an unacceptable infection risk (Jorgensen, 1977).

Capacitive Coupling is noninvasive which eliminates the constraints of operative placement of the device and the risk of infection. However, the fall off of the electrical field in tissue of high resistivity like skin and subcutaneous fat – ∼1/*r* ^2^ where r is the distance from the signal source – is prohibitive for treatment of many long bone fractures (Plonsey and Barr, 1995). This is reflected in the large animal literature utilizing CC to induce osteogenesis. One study demonstrated no effect with 5–10 mA at 60 kHz in a canine tibial distraction osteogenesis model (Pepper et al., 1996). However, a more recent study showed benefit at 1.5 mA, 12.5 Hz for sheep tibial delayed union by quantitative histomorphometry (*p* < 0.0001) and quantitative radiodensity analysis (*p* < 0.0043) (Muttini et al., 2014). An important note is that in this study, the electrodes were implanted in bone proximal and distal to the fracture and, for this reason, did not have to overcome the resistivity of skin and subcutaneous adipose tissue. Therefore, CC is not applicable to treatment of fractures outside of the spine, middle tibia, wrist, and ankle. Of the CC studies included, three studies (Benazzo et al., 1995; Beck et al., 2008; Piazzolla et al., 2015) describe administering currents transcutaneously that are orders of magnitude lower than the extremum of the dose-response curve modeled for CC. In a rat sciatic denervation osteoporosis model, Brighton et al. found that with constant voltage stimulation of 0.66 V (estimated current of 584.6 ± 21.2 μA) the dry weight of osteoporotic tibia was closest to the contralateral control (ratio of 0.922, *p* < 0.05) (Brighton et al., 1988). Scaling this dose-response curve to humans with thicker skin and significantly more interposing soft tissue between the skin and target bone, it is unlikely that administering 5 mA of stimulation would penetrate the skin, much less have any effect on bone.

Finally, PEMF appears to be an attractive option for treatment of acute fractures as it is both noninvasive, and the induced electrical field can more easily penetrate soft tissue. However, there are significant patient compliance issues with these devices that make them less practical. Many protocols required lengthy durations of treatment, resulting in low levels of patient compliance (Faldini et al., 2010). Other variables also significantly affect the induced electrical field. For example, the thickness of tissue planes (fat, muscle, and bone) and their relative difference in resistivity, as well as even small changes in the placement of the PEMF device with relationship to the fracture, dramatically change the intensity of the electrical field at the fracture site (Lunt, 1985). By contrast to *in vitro* and small animal studies, large animal studies have largely been unsuccessful in inducing osteogenesis with PEMF therapy (Nicksic et al., 2022). Studies to date have included a range of indications in both canine and sheep models (Miller et al., 1984; Law et al., 1985), but the single study that was able to demonstrate benefit for PEMF therapy applied 0.2 mT, 1.5 Hz stimulation to a canine mid-diaphyseal tibial 2 mm gap osteotomy model (Inoue et al., 2002). This stimulation was applied for 4 weeks for 1 h per day, beginning at 4 weeks post-injury. More work is required to determine the dose-response curve for PEMF therapy in large animals and humans.

In human studies, these issues are not completely addressed by cast-implanted PEMF devices, as this strategy adds new variables including the thickness and resistivity of the cast material and air between the cast and the skin. Unless the PEMF devices are calibrated on a patient-by-patient basis and compliance issues are addressed, the results of cast-implanted PEMF studies will remain unpredictable.

**FIGURE 4:**
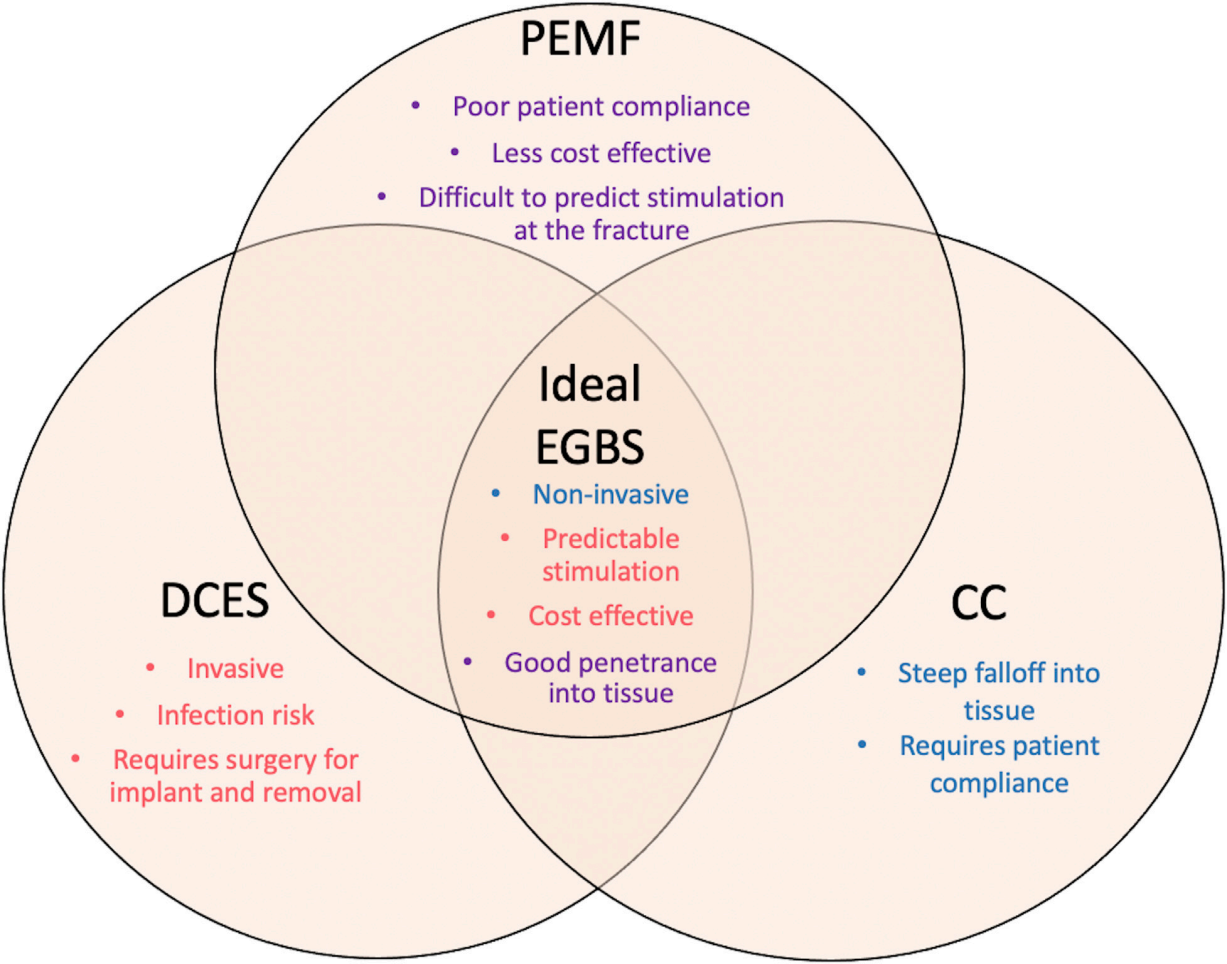
This diagram represents the qualities of the ideal electronic bone growth stimulator. The characteristics of the different modalities of ES are listed under each type. The weaknesses of each type of ES are listed on the periphery of the figure. The strengths of each type of modality that should comprise the ideal EGBS are listed centrally. Purple text denotes qualities of PEMF. Red text denotes qualities of DCES. Blue text denotes qualities of CC. This figure is meant to emphasize the need for innovation in the field of bone growth stimulation, as the ideal EGBS does not yet exist.

Based on the available literature, several patterns emerge that could potentially lead to an ideal EGBS. It should possess qualities of all the available modalities for ES (Figure 4). First, like DCES, the strength of stimulation should be predictable at the level of the fracture, regardless of thickness of intervening soft tissue or patient position. Second, similar to CC and PEMF, it should also be semi- or noninvasive so that there is negligible risk of infection, and the device can be administered non-operatively. Lastly, it should be cost effective so that it can be used widely. Further innovation beyond what is currently available, perhaps in the field of conductive microparticles, could potentially offer a viable and clinically relevant methodology of electrical stimulation for bone healing.

### 6.3 Future Directions for Electrical Stimulation

Outside of the indication for augmentation of acute fracture healing, there is a robust body of literature to demonstrate that ES can be used to improve peripheral nerve regeneration, neuroplasticity, and soft tissue wound healing (Zuo et al., 2020; Luo et al., 2021; Sousa et al., 2022). One key component of the literature for these fields is that the dose-response curve and optimal duration of therapy is well described. To date, these important elements are missing from the bone stimulation literature. For other osseous indications, treatment of osteoporosis has been largely unsuccessful in preclinical studies with ES therapy likely for reasons previously stated (i.e., electromagnetic fields are not uniform through space and efficacy of treatment is heavily dependent on stimulator placement) (van der Jagt et al., 2012). Before attempting to address systemic bone diseases, delineation of a clear dose-response curve and optimal duration of therapy should be achieved for focal osseous injuries.

## 7 Conclusion

Electrical bone growth stimulators have shown promise in treating acute fractures in preclinical studies. To date, there is no agreement as to their efficacy in humans. The next steps to studying ES for the purpose of healing acute fractures in humans are to select the appropriate study population, report replicable data for device specifications, and develop sound methodologies to minimize bias. In the same vein, development of a more practical and reliable modality of ES is needed to maximize outcomes.
